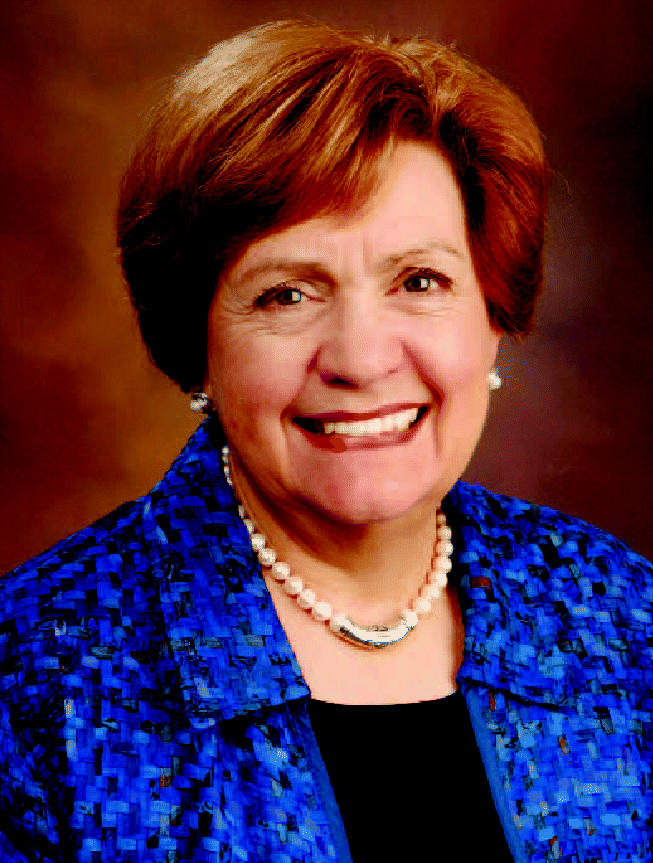# Anne Sassaman’s Farewell to the NIEHS Extramural Community

**Published:** 2006-11

Twenty years ago I arrived at NIEHS to direct what was then the Extramural Research Program. The staff was small and the portfolio consisted primarily of a collection of R01 grants and the Environmental Health Science and the Marine and Freshwater Biomedical Sciences Centers.

This month I retire as Director of the Division of Extramural Research and Training (DERT), which has grown in staff from three to more than 20 program officers and program analysts, with concomitant increases in review, grants management, and other administrative staff. More important has been the significant and exciting growth in the research and training programs supported by the NIEHS. Since 1998, we have used this space in *EHP* to communicate with readers about those programs and to provide staff contact information. I hope this has been an effective way of letting you know what is happening at NIEHS.

Please permit me a few lines to reminisce about some of these programs. The first major challenge I faced was the creation in 1986–1987 of the Superfund Basic Research and the Worker Education and Training Programs, which have grown from $3 million and $10 million respectively, to just under $51 million and $45 million in 2006. When Dr. Kenneth Olden became director of the institute in 1991, we grew both in terms of staff and new programs. Dr. Olden’s legacy was a redefinition of NIEHS from primarily a testing institute to a public health institute. During his tenure, the extramural programs were focused by the development of a number of solicited initiatives. Not only did we create new basic science programs, such as the Environmental Genome Program and the Toxicogenomics Research Consortium, but we became a leader in the areas of environmental justice, community-based participatory research, and K–12 education.

Another era in the history of NIEHS began when Dr. David Schwartz became the fourth director and immediately instituted a strategic planning process (http://www.niehs.nih.gov/external/plan2006/home.htm). Once again, DERT has been the focal point of developing new initiatives, such as the ONES program, the DISCOVER centers program, the Exposure Biology program, as well as exciting new funding opportunities in epigenetics and comparative genomics to support and implement the Strategic Plan and new vision.

I leave with a sense of pride in these accomplishments, which would not have been possible without an absolutely outstanding and dedicated staff. Leadership of such a fine group of professionals has been a pleasure, and I feel privileged to have been a part of NIH and NIEHS. I also want to offer my sincerest thanks to all who have served as advisors in many capacities, and especially to those who have made these programs successful by responding to our initiatives and performing the research.

Good luck in the future.

Anne P. Sassaman, Ph.D.

## Figures and Tables

**Figure:**